# An updated study protocol for a real-life digital 12-month weight management program, the Healthy Weight Coaching

**DOI:** 10.1080/07853890.2024.2396562

**Published:** 2024-09-04

**Authors:** Aila J. Ahola, Anu Joki, Laura U. Suojanen, Kirsi H. Pietiläinen

**Affiliations:** a Obesity Research Unit, Research Program for Clinical and Molecular Metabolism, Faculty of Medicine, University of Helsinki, Helsinki, Finland; b Healthy Weight Hub, Abdominal Centre, Helsinki University Hospital and University of Helsinki, Helsinki, Finland; c Research Program for Population Health, Faculty of Medicine, University of Helsinki, Helsinki, Finland

**Keywords:** Digital weight loss intervention, protocol, obesity

## Abstract

Obesity is an important health concern that poses many public health challenges. Evidence-based treatment modalities, capable of cost-effectively reaching large patient groups are needed. In this paper, we present the design and methods of the updated national, 12-month, digital weight management program, the Healthy Weight Coaching (HWC). The major updates, as compared to the previous version, are related to the theoretical background of the obesity management and updated BMI cut-offs. The HWC is available, based on physicians’ referrals, to adult Finnish citizens with BMI ≥30 kg/m^2^ or ≥27 kg/m^2^ with a comorbidity, who have a health-based need to lose weight. Rooted in the principles of behavioural therapy, the HWC focuses on teaching coping skills, guides to healthy self-reflection, and supports concrete lifestyle changes as part of healthy weight loss. The automated weekly training sessions, supplemented by 3-8 exercises, form the basis of the program. These sessions address topics such as diet, physical activity, stress management, and rest and recovery. Additionally, a personal coach is allocated to each patient to provide tailored support. At baseline, patients record their weight, height, and waist circumference, online, and complete questionnaires on lifestyle, diet, physical activity, sleep, psychological factors, and health. Thereafter weight recording is conducted at least every 4 weeks, while the questionnaires and measuring the weight circumference are repeated at 3, 6, 9, and 12 months. In addition, patients can make use of diaries and peer group chats for additional support. Data collected from the consenting patients will be used for research purposes with the weight change from baseline to 12 months as the main outcome in the real-life observational study. The study will provide invaluable insights into the application of digital modalities in the treatment of obesity.

## Introduction

Obesity remains a critical health concern, persistently posing significant challenges to public health and well-being. According to the recent estimates, by the year 2035, approximately every fourth individual globally exceeds the threshold of obesity (Body Mass Index, BMI ≥30 kg/m^2^), while every other individual is classified with at least overweight (BMI ≥25 kg/m^2^) [1]. The burden related to excess body fat extends from potential individual nuisance and suffering to various comorbidities [2, 3], reduced working ability [4], and increased mortality [5], which also impact the society at large.

As obesity tends to become a chronic condition, its prevention is of upmost importance. Once developed, treating existing obesity has shown to reduce the risk of type 2 diabetes [6] and it likely also improves cardiovascular health [7]. The traditional face-to-face obesity treatment is costly, however, and may not be available for a large proportion of the population in need. To tackle with the increasing demand of obesity management, digital modalities, that have the potential to cost-effectively treat large patient groups, have been developed. Importantly, web-based interventions are more effective than control interventions or no care and are comparable, in their efficiency, to face-to-face interventions [8].

Amongst the digital obesity treatment modalities, developed over the past years, is the Healthy Weight Coaching (HWC) [9, 10]. The HWC is a 12-month interactive program, provided by the Abdominal Center of the Helsinki and Uusimaa Hospital District *via* a web-based platform, the HealthyWeightHub.fi [11]. The HealthyWeightHub.fi is part of the Health Village (Terveyskyla.fi) [12], a digital portal, coordinated by the Helsinki University Hospital and developed in collaboration between the five university hospital districts and several patient associations, in Finland. The virtual service was developed to complement the traditional, face-to-face, treatment pathways. Of the virtual houses, the HealthyWeightHub.fi was designed to provide evidence-based, accessible, affordable, and cost-effective obesity treatment for Finnish citizens in need of weight loss support. In addition to the two digital treatment programs, one for conservative treatment (HWC) and one for those proceeding to surgical treatment of obesity, the HealthyWeightHub.fi contains a public domain, with general information and self-help instructions for weight management, and the Health Village PRO, a service designed for the professionals in the social welfare and health care sectors (Table 1). In this paper we focus on the conservative treatment arm, the HWC, of the HealthyWeightHub.fi.

**Table 1.** HealthyWeightHub.fi service.

Public domain	Healthy Weight Coaching	Surgical obesity management	Health Village PRO
ABCs on obesity and weight loss	Based on referral	Based on referral	For professionals in social welfare and health care sector
Videos and audios	Virtual personal coach	For those aiming at bariatric surgery	Online courses
Tests	52 weekly sessions		Digital tools
Self-help instructions	3-8 trainings per session		Guides
8-week self-help intervention	Videos and audios		
Guided self-help programs	Informed consent for research purposes		

The HWC was first launched in October of 2016 and the original treatment protocol, which was based on the Acceptance and Commitment Therapy, was published by Suojanen et al. [9]. In our preliminary results of the original protocol, we observed a mean weight loss of 4.6%, in those reaching the 12-month timepoint, and 43% of the completers achieved clinically relevant weight loss (≥5%) [10]. Based on accumulated scientific evidence, gained experiences, and user feedback, the service is continuously developed. In this paper, we present the design and the methods of an updated treatment protocol, launched in March 2024. Key novelties and improvements include revised eligibility criteria, an expanded theoretical foundation incorporating multiple therapeutic approaches, the integration of the latest scientific knowledge on the biology of obesity and weight loss, and a stronger emphasis on reducing obesity-related stigma.

## The Healthy Weight Coaching

Patients enter this real-life weight management program *via* referrals from any licenced physician, in Finland. The service is nationally available, and the costs are covered by the patient’s municipality of residence. The service is available both in Finnish and Swedish, and it can be accessed *via* web browser and mobile application. The 12-month program aims at holistic and sustainable life style changes that support long term weight management. Integrating various orientations from behavioural therapy, including cognitive therapy, acceptance and commitment therapy, solution focused therapy, and psychodynamic therapy, the HWC focuses on teaching coping skills, guides to healthy self-reflection, self-compassion, and supports concrete lifestyle changes as part of a healthy, gradual weight loss and subsequent weight maintenance. As disordered eating is prevalent in obesity, the program prioritises normalising eating behaviours to support overall health.

### To whom

The HWC is a referral based, nationwide program targeted for adult (18-70 years) individuals with overweight or obesity (BMI ≥30 kg/m^2^ or BMI ≥27 kg/m^2^ with some comorbid condition), who require weight management assistance, as determined by the referring physician. Prior to being accepted to the program, inclusion and exclusion criteria are reviewed from the referral documentation provided by the healthcare provider. The patient must have a favorable life situation and sufficient resources to commit to independent and long-term change efforts. The patient must have sufficient verbal and written language skills, as the program is currently available in Finnish or Swedish. As the service is digitally provided, it requires a computer or a mobile device with internet access, and at least some computer literacy. Disorders that substantially impact cognitive skills, severe depression or other acute psychological conditions, cancer or some other serious illnesses, and pregnancy are contraindications for the program. The patient should not have an acute diagnosed eating disorder. However, many individuals with obesity having been included in the program may have undiagnosed eating disorders. If eating disorder symptoms significantly impair progress, the team may refer the patient to a more suitable treatment program.

Individuals may also enrol to the program as part of the preparation for a planned metabolic bariatric surgery. Here, should the patient achieve the required 5% weight loss by the six-month time point, they may be referred for surgical treatment, including a switch to the HealthyWeightHub.fi surgical arm.

### Weekly training sessions

The automated weekly training sessions, addressing weight management-related topics such as diet, physical activity, sleep and recovery, psychology, and overall health, form the basis of the program (Table 2). There are a total of fifty-two sessions of 30-60-minutes’ duration which, if completed with a frequency of one session per week, will take a year to finalize. However, once a given session has been completed, access to the next session is directly available. This enables one to complete the HWC even in a shorter period of time. Throughout the 52 sessions, the topics are explored from various angles, with themes deepening as the program progresses, helping the patient to further practise and reinforce their knowledge around the themes. Moreover, topics related to the maintenance of the adopted life style changes are incorporated towards the end of the program to prepare the patients for transitioning out of the program and sustaining the acquired skills. While the weekly sessions provide information about weight- and weight loss-related topics, the program steers away from one-size-fits-all approach. Complemented by 3-8 weekly exercises, the program aims at cultivating mindfulness skills and fostering deeper understanding of one’s health and its interconnected factors. To further support these aims, informative videos, audios, and links are incorporated to provide variety in the treatment modalities and to increase motivation. Patients are encouraged to integrate the themes, covered during the weekly sessions, in their daily lives and, at the beginning of each session, to reflect their experiences and thoughts related to the themes covered during the previous session.

**Table 2.** Main contents of the Healthy Weight Coaching.

Content	Core ideas
Goal setting	Setting realistic goals and planning how they can be achieved. Monitoring the achievements during the program.
Healthy diet	Information about the healthy diet and dietary recommendations for weight loss and maintenance. Plate model, portion sizes, sufficient intake of protein, vegetables, and fibre, and selecting healthy snacks are discussed and practiced. MealLogger can be used to record and monitor the actualized food choices.
Hunger and satiety	Recognizing hunger and satiety are acknowledged as central themes in weight maintenance. Guidance on how satiety can be boosted is provided.
Physical activity	The importance of regularity in physical activity. Tools how to increase physical activity, at all levels, are provided.
Sleep and recovery	The role of sleep and recovery in weight loss. Patients are encouraged to practice good sleep hygiene.
Thoughts and mindfulness	Patients practice recognizing their thoughts and their impacts on wellbeing. Mindfulness exercises are provided.
Stress	The role of stress in overall health and weight management. Practices to reduce stress are conducted.
Values	Patients are encouraged to identify one’s most important values and to live their lives according to values that best support their overall wellbeing.
Weight management	Information and practices on weight management are provided (e.g. appetite control, eating frequency, planning skills, making healthy food choices, shopping for food)

### Personal coach

In addition to the automated program, a health care professional, either a registered nurse, licenced dietitian, physiotherapist, or psychologist, is appointed to each patient to serve as a personal coach. At the beginning of the program, one phone call is organized between the patient and the coach. The aims of this introductory phone call are to build rapport and to formulate the goals and the means by which these goals can be met, with the patient. Following this initial contact, all communication takes place digitally. During these one-on-one online discussions, the coach can provide tailored support and suggest specific exercises based on patient’s individual needs. On average, we have observed a mean of 40 contacts between the patient and the coach, during the program. In addition to the one-on-one messaging, the coach periodically organises group chats among the patients. During these voluntary chats, the patients may anonymously exchange their experiences, and provide and receive peer support.

### Diary

Patients are encouraged to use digital diaries that are integrated into the program. Using the diaries may help the patients to better track the changes that take place during the weight loss journey. In these diaries, patients may record their weekly plans, goals, feelings, physical activity, weight, and waist circumferences. The coaches have access to these diaries and may use the provided information to support the patient during the program. For example, a typical diary entry might include setting weekly goals such as increasing physical activity to 30 min per day, documenting feelings related to cravings or progress, and recording measurements of weight and waist circumference.

### The MealLogger

In order to keep track of one’s food intake, the patients may take advantage of the MealLogger application [13]. The MealLogger is an electronic food diary where the patient enters time-stamped photographs of their meals, include a detailed description of them, and identify the type of the meal (breakfast, lunch, dinner, snack, or beverage) consumed. Using the app may help the patient to assess their food consumption more objectively and to track any changes that may occur during the process. Those interested, may also take part in quarter-yearly MealLogger groups where the pictures and food ideas can be shared with other group participants. Moreover, when agreed with the patient, the coach can provide their feedback on the meal pictures.

### Very low calorie diet in the Healthy Weight Coaching

Patients with a BMI >30 kg/m^2^ who, based on the referring physician’s assessment, could gain health benefits from it, may take part in a maximum of a 10-week very low calorie diet (VLCD) period, during the HWC. If incorporated into the program, the VLCD is conducted in alignment with the patient and under dietitian’s guidance. Before initiating the VLCD, the need to make any modifications to the prescription medication, is assessed. Moreover, as severe dietary restrictions can trigger binging episodes in some individuals, the binging symptomatology is evaluated beforehand and, when needed, discussed with the patient. Based on our prior experience, only a small proportion of patients take part in the VLCD. In the study reports, the VLCD component is taken into account in stratified analyses and accounting for VLCD use in the statistical analyses.

### FAQ and additional training assignments

In the Frequently Asked Questions -section, some of the most important topics related to the HWC are collected. Included are themes such as how to contact the coach, dietary recommendations, suggestions how to increase physical activity, how to access MealLogger, the importance of rest and recovery, and information about the group chats. FAQ also serves as a depositary for the weekly plan template, podcasts, exercise programs, and other extracurricular training material, to which the coaches can refer the patients when there is interest in additional training assignments.

### Automated reminders

To provide information and to improve adherence, the program sends automatic e-mail- and/or SMS-reminders to the patients. These messages remind the patient of the importance of registering or logging-in to the program in case of sub-optimal activity, provide information about patient’s progress in the program, and notify of the incoming messages and group chat invitations.

### Terminating the program

Generally, the patient’s participation, in the HWC, is automatically discontinued at the 1-year time-point. Importantly, in case of any major health or personal reasons, the program can be put on hold for a maximum of two-months. Any such prearranged break will subsequently prolong the program from the end. However, should the patient show no advancement in the weekly sessions for more than 2 consecutive months without communicating this to the coach, the participation is automatically discontinued. Once the program participation has reached its end, the patients continue to have access to the program to review their prior entries but will not be able to complete any new exercises. However, the MealLogger application will be available and fully functional even when the patient is no longer in the HWC.

### Differences between the original and updated protocol

In the previous version of the protocol [9], patients with a BMI ≥25 kg/m^2^ were accepted. The updated protocol now accepts individuals with a BMI ≥30 kg/m^2^, or those with a BMI ≥27 kg/m^2^ with a comorbid condition. A significant enhancement in the current version is the expansion of its theoretical foundation. While the previous protocol focused solely on acceptance and commitment therapy, the updated program now integrates a diverse range of behavioral therapies, including also cognitive therapy, solution-focused therapy, and psychodynamic therapy.

Moreover, the updated program incorporates the latest scientific insights into the biological foundations of obesity and weight loss, such as regulation of appetite, satiety, energy expenditure and the role of sleep. An additional key improvement is the increased emphasis on reducing obesity-related stigma, ensuring a more comprehensive and supportive approach to weight management.

## Scientific study

Upon entering the HWC, patients may provide their informed consent to have their data included in scientific research. The main aim of the study will be to investigate the effectiveness of the digital HWC in treating overweight and obesity. Importantly, in order to provide evidence-based weight loss interventions, also in the future, we need to investigate if costly face-to-face treatment is needed or whether digital coaching can induce comparable or even superior results. Moreover, in order to further develop the program, we will investigate the utilisation rates of the different exercises and treatment paths, in the program, and their effects on the weight loss. Variables available for the study are listed on Table 3. The data are mainly collected using online questionnaires, which are completed at baseline and at 3-, 6-, 9-, and 12-month timepoints. However, data on age, sex, and municipality are derived from national register, since the patients log in to the program using a secured national personal identification system. The Ethics Committee of Helsinki and Uusimaa Hospital District (the 4^th^ EC panel) approved the study protocol (327/13/03/00/2015, GDPR update 587/2019). All procedures performed in the study will be conducted in accordance with the ethical standards of the institutional research committee and with the 1964 Helsinki declaration and its later amendments or comparable ethical standards.

**Table 3.** Study variables.

Variable type	Variable	Method
Socio-demographic variables	Age	From national registry
	Sex	From national registry
	Municipality	From national registry
Anthropometric measurements	Weight	Self-measured
	Height	Self-reported from health records or self-measured
	Waist circumference	Self-measured
Health	Diseases	Self-reported
	Medication	Self-reported
	Pain	Self-reported frequency, intensity, site of pain
	Use of healthcare services	Self-reported health care visits
Other weight loss methods used	Metabolic bariatric surgery	Patient records
	Use of weight loss medication	Self-reported
	Use of very low calorie diet	Self-reported. Recommended in the referral and final decision by the patient and the coach
	Perception of the causes of weight gain	Self-reported factors that have contributed to weight gain
Psychological variables	Motivation to change	Self-reported willingness to behavioural change
	Stigma	Self-reported experiences of bullying and stigmatisation due to weight
	Life satisfaction	Self-reported satisfaction of life, personal life circumstances, and relationships
	Mental resources and burdensomeness	Self-reported personal strength and resilience
	Quality of life	EuroHIS-8
Lifestyle	Sleep	Self-reported amount and quality of sleep, tiredness
	Physical activity	Self-reported frequency, time, and intensity
	Eating behaviour	Self-reported eating patterns: control of eating, portion size, satiety, snacking, cravings, emotional eating
	Binge Eating Scale -questionnaire	BES
	Alcohol consumption	Audit-C
	Smoking and nicotine products, cannabis	Self-reported history, current use frequency and, amount

BES, Binge Eating Scale; Self-reported, the data are self-reported in a non-validated online questionnaire.

### Anthropometric measurements

Upon entering the HWC, patients report their weight, height, and waist circumference on a web-based form. Patients are instructed to measure their weight, using patients’ domestic scales, in the morning immediately after using the restroom. Patients report their previously recorded height, but if they are unsure, they are instructed to measure it without shoes, standing straight against a wall. They are also guided to measure their waist circumference at the narrowest point between the ribs and hips. As part of the training sessions, weight is recorded every 4 weeks. Following baseline, waist circumference is reported every 3-months. In addition, patients are encouraged to regularly, for example on a weekly basis, record their weight on an online diary. The online diary provides patients with a graphical representation of the weight for easy tracking. For the study, the BMI is calculated based on the self-reported height and weight. To provide comparable measurements among the participants, interpolated weights representing the 3-, 6-, 9-, and 12-month timepoints will be used as a basis for the BMI-calculations.

### Data on participation activity

Patients’ activity in the program can be assessed using the log-in data and by examining how many and which exercises were conducted. Furthermore, the numbers of weight recordings and completed weekly training sessions, use of diaries, and the frequency of patient-coach communication can be used as an estimate of the participation activity. In the analyses, the role of patient activity in weight loss success can be examined.

### Outcomes

The primary outcome is the percentage of weight change from baseline to 12 months. Moreover, weight loss at 3-, 6-, and 9-month timepoints, as compared to the baseline, will be assessed. In addition to the continuous weight change percent, we will also investigate the categorical weight loss, defined as the percentage of individuals reaching clinically relevant weight loss, and the changes in waist circumference. Whether other predictors, such as socio-demographic, health, psychological, or lifestyle factors impact the weight loss success, will also be investigated. Beyond weight loss, we are also interested in the effects of the program participation on diseases, medication use, pain, healthcare resource utilisation, motivation, life satisfaction, mental resources, burdensomeness of life, quality of life, sleep, physical activity, and eating behaviour.

### Statistical analyses

Basic characteristics will be reported as frequencies for categorical variables, means ± standard deviations for continuous variables that follow normal distribution, and medians (interquartile ranges) for continuous variables with skewed distribution. For these respective variables, between-group-differences will be investigated using Chi-squared test, independent sample’s t-test, and Mann-Whitney U-test. For investigating the independent associations between variables of interest and continuous and categorical weight loss, as outcomes, linear and logistic regression analyses, respectively, will be applied. For identifying different weight development patterns, we will cluster the continuous weight data using time series clustering methods (e.g. hierarchical clustering with the dynamic time warping metrics). Factor analysis can be used for identifying underlying constructs from the collected data. When relevant to the research question, mediational analysis can be applied. Moreover, we will apply machine learning algorithms to develop individualized models to predict the expected treatment response based on weight and baseline characteristics.

## Discussion

In this paper, we introduce a protocol for an updated real-life weight management intervention, the HWC, within the HealthyWeightHub.fi portal. With the vastly increased rates of obesity, there is a great need for effective, evidence based, and cost-effective methods to treat patients struggling with excess weight. As a digitally implemented and publicly funded healthcare service, the HWC provides the patients with care regardless of their place of residence or financial status, endorsing treatment equality among the citizens. Moreover, the around-the-clock accessibility ensures that the service is readily available whenever required.

Several reviews evaluating the effects of digital weight management programs have been published, showcasing a range of outcomes in terms of weight loss success. Allen et al. for example, observed that 48% of the web-based randomized controlled trials demonstrated statistically significant weight loss as compared to control groups [14]. Kodama et at. reported that web-based lifestyle modification interventions exhibited modest but significant additional weight loss effect compared with control groups not using Internet [15]. Moreover, while integrating Internet into conventional obesity care proved effective, using it as a substitute for face-to-face support yielded less favourable outcomes [15]. In their review of 20 systematic reviews, Sorgente et al. found web-based weight management interventions superior to minimal treatments [16]. However, compared to face-to-face interventions, web-based weight loss interventions were less effective [16]. Finally, covering 26 systematic reviews not included in the above papers, Kupila et al. found eHealth interventions more effective than control interventions or no care and comparable to face-to-face interventions [8].

Importantly, the observed effect sizes remain relatively small in studies comparing eHealth interventions to control conditions, suggesting that, despite their potential statistical significance, the obtained results may not always be clinically significant. However, besides weight change, comprehensive health interventions such as HWC have the potential to induce also other beneficial changes including improvements in overall health, eating behaviour, and physical activity, reduction in health care resource utilization, and enhancements of life satisfaction and quality of life. Making meaningful comparisons among studies that greatly differ in their patient characteristics, sample sizes, methods of delivering the program, and program durations can be challenging. Regardless, several components that improve the success in digital weight loss interventions have been identified, including goal setting, self-monitoring, coaching, encouragement, tailored content, group support, use of a structured program, and professional feedback. Crucially, all the aforementioned components have been integrated into the HWC program, to ensure the best possible support for patients while maintaining the program within a virtual environment.

There are several strengths and limitations related to the study. Among the strengths is the use of data collected from of a large, national sample of individuals taking part in a real-world prospective weight management program. Real-world studies are currently under-represented in the scientific literature of digital weight loss programs and, while the randomized controlled trials are the gold standard to demonstrate efficacy, more evidence is needed on how the programs work in a real-life setting with diverse patient populations. A real-life cohort study conducted within a clinical practice setting has the potential to improve the generalizability of the findings. Moreover, as a national endeavour, the HWC is also likely to attract a large patient sample, which can enhance the reliability of the results. To ensure the validity of the effectiveness estimates, we will also measure and account for dropouts and other factors that could impact the study’s validity. Limitations include the lack of control group, which is explained by the real-life study design. Other important limitations are the use of self-measured anthropometric data and self-reported questionnaires. It is important to recognize, however, that the HWC primarily serves as a real-life obesity management program. Consequently, the selected methods, including self-reporting, are tailored to this context and may not strictly adhere to the most rigorous scientific research standards. Nevertheless, the research conducted using data collected from the HWC will provide a significant contribution to the scientific body of knowledge regarding the use of digital weight management methods in curbing an important global health issue.

In conclusion, the HWC is a national, digital, multi-component weight management program designed to support adult patients with overweight or obesity in making long-term behavioural changes and thereby promoting health and weight loss. The data collected from consenting individuals, taking part in this prospective real-life endeavour, will be used in scientific research. The study will provide invaluable insights into the application of digital modalities in the treatment of obesity. The observations may also be used to inform future interventions, shaping the landscape of obesity management, and paving the way for more effective treatment.

## Data Availability

Due to the nature of this research, participants of this study did not agree for their data to be shared publicly, so supporting data is not available.
